# Implementation of mifepristone medical abortion in Canada: pilot and feasibility testing of a survey to assess facilitators and barriers

**DOI:** 10.1186/s40814-019-0520-8

**Published:** 2019-11-08

**Authors:** Courtney Devane, Regina M. Renner, Sarah Munro, Édith Guilbert, Sheila Dunn, Marie-Soleil Wagner, Wendy V. Norman

**Affiliations:** 1 Contraception and Abortion Research Team, Women’s Health Research Institute, BC Women’s Hospital and Health Center, Vancouver, BC Canada; 2 School of Nursing, University of British Columbia, Vancouver, BC Canada; 3 Faculty of Medicine, University of British Columbia, Vancouver, BC Canada; 4 Institut national de santé publique du Québec, Quebec City, QC Canada; 5 Department of Family and Community Medicine, University of Toronto, Toronto, ON Canada; 6 Department of Obstetrics and Gynaecology, University of Montreal, CHU Sainte-Justine, Montreal, QC Canada; 7 Faculty of Public Health & Policy, London School of Hygiene and Tropical Medicine, London, UK; 8 Department of Family Practice, Women’s Health Research Institute, University of British Columbia, E202-4500 Oak Street, Vancouver, BC V6H 3N1 Canada

**Keywords:** Mifepristone, Canada, Abortion, Survey, Family planning, Family physician, Delphi approach, Diffusion of Innovation, Implementation science

## Abstract

**Background:**

Direct primary care provision of first-trimester medical abortion could potentially address inequitable abortion access in Canada. However, when Health Canada approved the combination medication Mifegymiso® (mifepristone 200 mg/misoprostol 800 mcg) for medical abortion in July 2015, we hypothesized that the restrictions to distribution, prescribing, and dispensing would impede the uptake of this evidence-based innovation in primary care. We developed and pilot-tested a survey related to policy and practice facilitators and barriers to assess successful initiation and ongoing clinical provision of medical abortion service by physicians undertaking mifepristone training. Additionally, we explored expert, stakeholder, and physician perceptions of the impact of facilitators and barriers on abortion services throughout Canada.

**Methods:**

In phase 1, we developed a survey using 2 theoretical frameworks: Greenhalgh’s conceptual model for the Diffusion of Innovations in health service organizations (which we operationalized) and Godin’s framework to assess the impact of professional development on the uptake of new practices operationalized in Légaré’s validated questionnaire. We finalized questions in phase 2 using the modified Delphi methodology. The survey was then tested by an expert panel of 25 nationally representative physician participants and 4 clinical content experts. Qualitative analysis of transcripts enriched and validated the content by identifying these potential barriers: physicians dispensing the medication, mandatory training to become a prescriber, burdens for patients, lack of remuneration for mifepristone provision, and services available in my community. To assess the usability and reliability of the online survey, in phase 3, we pilot-tested the survey for feasibility.

**Results:**

We developed and tested a 61-item Mifepristone Implementation Survey suitable to study the facilitators and barriers to implementation of mifepristone first-trimester medical abortion practice by physicians in Canada.

**Conclusions:**

Our team operationalized Greenhalgh’s theoretical framework for Diffusion of Innovations in health systems to explore factors influencing the implementation of first-trimester medical abortion provision. This process may be useful for those evaluating other health system innovations. Identification of facilitators and barriers to implementation of mifepristone practice in Canada and knowledge translation has the potential to inform regulatory and health system changes to support and scale up facilitators and mitigate barriers to equitable medical abortion provision.

## Background

Abortion health services are safe, common, and legal in Canada [1]. The rate of induced abortion is approximately 14–15/1000 females aged 15–44, annually [2, 3]. In Canada, abortion is a publicly funded service. Abortion services are accessed in Canada either by self-referral to an abortion/reproductive health care facility or through a referral from their family physician to an abortion provider [4, 5]. In 2016, surgical terminations represented 94.7% of all first-trimester abortions across the country [2]. In Canada, geography is a significant barrier to accessing abortion facilities; for example, in the province of British Columbia, 90% of all abortions are provided in large urban cities, despite the fact that 43% of reproductive-aged women live outside these metropolitan areas [6]. Having abortion services limited to urban centers significantly impacts access to care for almost half of the population of reproductive-aged women in Canada [4].

One solution to increase equity in access to abortion is through the provision of first-trimester medical abortion in primary care settings. In 2005, the World Health Organization (WHO) added mifepristone (RU 486) to their list of essential medications, naming mifepristone the gold standard for first-trimester medical abortion care [7]. In high-income countries, the implementation of mifepristone has been associated with an increase in the proportion of abortions performed as medical abortions [8–11]. However, rates of mifepristone implementation have differed greatly internationally [9, 10, 12–14].

Emerging evidence from Australia and the USA suggests that variations in access to mifepristone may be due to the differences in health systems, provider training and supports, financial barriers, and legal restrictions [15–17]. For example, in the USA, mifepristone may be dispensed only by the prescriber and not by a pharmacist. Ten years after the introduction of mifepristone in the USA, first-trimester medical abortions only represented 36% of all abortions compared to over 80% of first-trimester abortions in many European countries [10, 18]. Furthermore, in Australia, where pharmacists may dispense mifepristone, women must pay $560 AUD upfront and attend a mandatory follow-up visit 1 week later [17], and abortion remains legally restricted or prohibited in some jurisdictions [19–21].

In July 2015, Health Canada approved Mifegymiso® (a combination product containing mifepristone 200 mg and misoprostol 800 mcg) for medical abortion, making Canada the 63rd country to approve the medication since 1988 [22, 23]. In 2016, the Society of Obstetricians and Gynecologists (SOGC) published new Canadian clinical practice guidelines for medical abortion [24], prior to commercial availability of the product. However, the initial approval of mifepristone in Canada stipulated restrictive constraints that did not conform with the clinical guidelines or usual clinical practice including distribution of the medication only directly to physicians and pharmacists who had completed a certified training program and registered with the licensed distributor, prescribing and dispensing limited to these certified physicians, ultrasound required for gestational dating prior to administration, physician observation of patient ingestion of mifepristone, and use up to only 49 days gestational age [22]. These stipulations were not consistent with the extensive evidence on safety and effectiveness for this medication. Family planning experts deemed the restrictions unnecessary [24, 25].

We hypothesized that these federal regulations would restrict patient access by impeding the uptake of mifepristone abortion practice, particularly for those professionals working in primary care, such as family physicians who are not primarily abortion providers, pharmacists, nurse practitioners, and midwives who have a critical role to play in expanding access to abortion services beyond large urban settings. We were unable to identify a survey instrument that comprehensively explored factors influencing the implementation of medical abortion practice. Prior to January 2017 when mifepristone became available in the Canadian market, we developed and pilot-tested a survey to investigate health policy, health system, and health care delivery factors that influenced the uptake and implementation process for mifepristone first-trimester medical abortion practice. Our objectives were to develop a survey instrument informed by a theoretical framework (phase 1), validate the survey content using qualitative group interview data (phase 2), and pilot test the usability and readability of the survey in an online format for feasibility (phase 3). Our goal was to understand how policy and practice characteristics relate to access to abortion services throughout Canada.

## Methods

We conducted this Mifepristone Survey Development Study as one component of our larger mixed-methods study, *Mifepristone Implementation Research in Canada* [26]*.* This comprehensive study examines the health policy, system, and service facilitators and barriers affecting the initiation and ongoing provision of medical abortion services in Canada [26]. Our data collection for the mixed methods study includes the following: (A) surveys with pharmacists and prescribers at baseline, 6 months and 1 year following the introduction of mifepristone medical abortion to their practice; (B) interviews with pharmacists and prescribers; (C) interviews with policy and health system stakeholders; (D) creation of an online community of practice platform to detect and support policy, system, and practice challenges; and (E) the evaluation of continuous integrated knowledge translation with health policy, system, and service decision-makers and health professional organizations (i.e., co-production of evidence by researchers and decision-makers). In this paper, we report the development process for the prescriber’s survey (data collection method “A”).

Our survey development process involved three phases that occurred between July 2015 and January 2017: (1) development of preliminary survey items [group I], (2) content validation via a modified Delphi process with a panel of physician experts [group II], and (3) pilot testing of the draft survey for reliability and usability in an online format [group III]. In phase 2, the panel of physician experts also provided their perceptions of the impact of facilitators and barriers on abortion services throughout Canada which further informed the content we aimed to cover in our survey instrument. This study was conducted by the members of the Contraception and Abortion Research Team—Groupe de recherche sur l’avortement et la contraception (CART-GRAC), a national, interdisciplinary, cross-sectoral collaboration engaging in research to support the provision of health services and implementation of policies that ensure equitable access to high-quality family planning knowledge, methods, and services for women and families throughout Canada [27]. Ethics approval for this study was obtained from the University of British Columbia Children’s and Women’s Hospital Research Ethics Board for research involving human subjects (H15-03207).

### Phase 1: Development of preliminary survey items

**Fig. 1:**
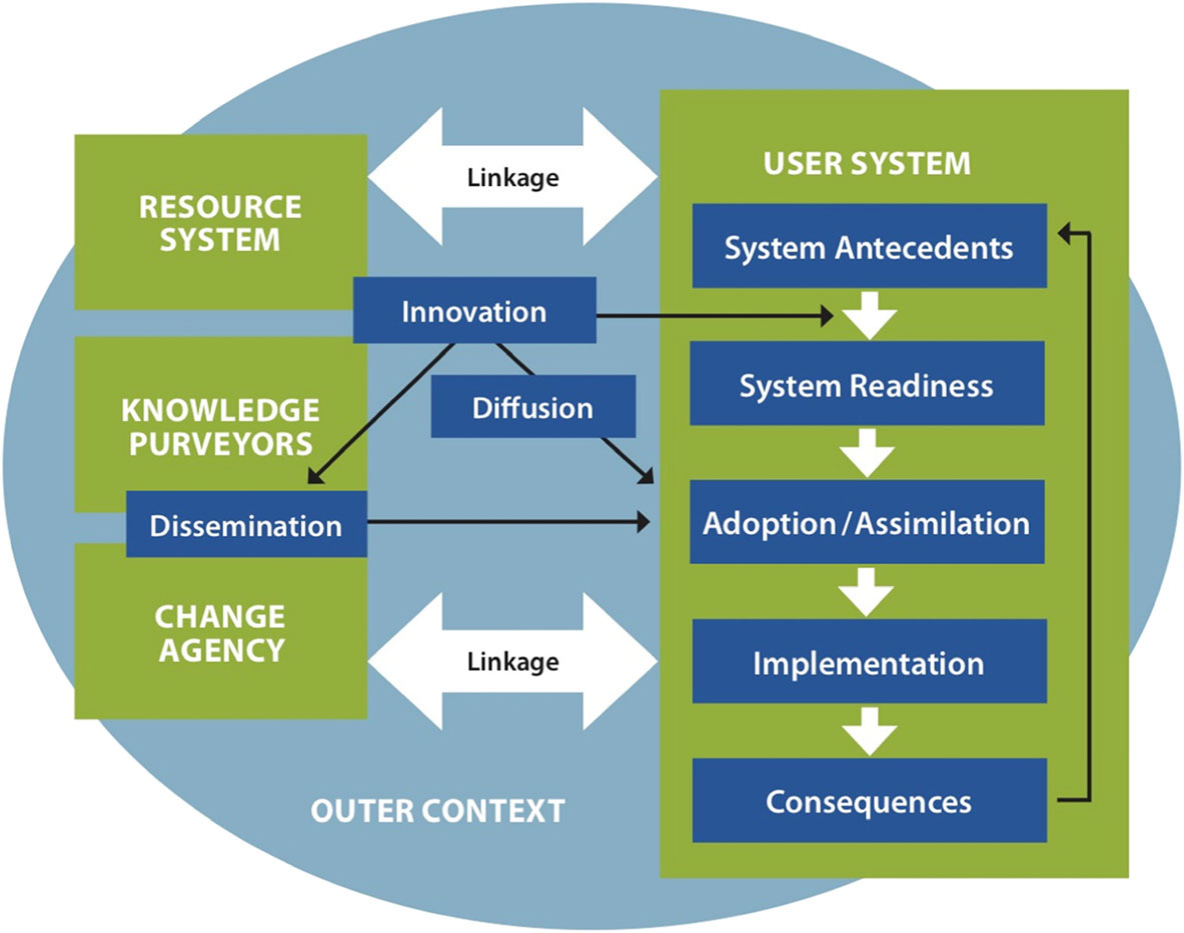
A conceptual model of diffusion of innovations in health service delivery and organizations, adapted from Greenhalgh et al. [28]

The development of the survey question constructs was guided by two conceptual frameworks: (1) *Roger’s Theory of the Diffusion of Innovation* as conceptualized by Greenhalgh et al. [28], which describes the determinants for implementation of innovations in health service delivery and has been used previously in Canada to understand the implementation of task-shifting contraceptive care from physicians to nurses [29]; (2) *Godin framework* [30], which integrates the Theory of Planned Behavior [31] and Triandis’ Theory [32] to predict an individual’s intention and uptake of clinical behavior. Greenhalgh’s framework posits that the interaction between the innovation, the intended adopter(s), and a particular context influences diffusion, dissemination, and implementation. The Greenhalgh framework consists of nine constructs: characteristics of the innovation and adopter, methods of diffusion and dissemination (e.g., communication and influence), system antecedents and readiness, outer context (e.g., external influences), resource systems and change agents (e.g., administrators, public health agents, public advocates), and the role of these constructs in facilitating the implementation process [28]. Légaré’s validated instrument [33] is based on the Godin framework [30] and assesses the impact of professional development on the uptake of new practice by measuring the following constructs: belief about capabilities, social influences, and beliefs about consequences, moral norms, and intentions. This framework has good application to practice in the abortion context, where role identity, moral norm, and social factors could have a strong influence on behavior. Figure 1 identifies these constructs and the dimensions by which each construct can be evaluated for the innovation’s adoption within the health system. Because mifepristone is a new approach to medical abortion services in a Canadian context, Godin and Greenhalgh’s frameworks fit conceptually with the goals of our survey.

Based on the conceptual frameworks above and previous work [34, 35], in October 2015, 4 of our clinician-researchers who are experts in abortion care (group I: RR, WVN, SD, EG) created a survey instrument with 47 baseline questions and 21 follow-up questions. The questions were developed to assess facilitators and barriers to the adoption of mifepristone into practice as well as the impact of mandatory training on the practice of mifepristone medical abortion. This occurred prior to the January 2017 market availability of mifepristone. The clinician-researchers identified key questions in a recursive fashion until consensus was reached. Constructs were restricted based on the relevance to abortion practice in Canada. The divisions of questions among Greenhalgh’s domains were as follows: Outer Context (*n* = 11), System Antecedents for Innovation (*n* = 6), Characteristics of the Innovation (*n* = 10), System Readiness for Innovation (*n* = 7, including 3 “matrix-style” questions with 32 response boxes combined), Adopter/Assimilation (*n* = 6), and Communication and Influence (*n* = 11) plus 15 demographic questions and 2 open-ended questions. An example of how questions were mapped to each domain of Greenhalgh’s conceptual framework is depicted in Table 1. Two Greenhalgh domains, Characteristics of the Innovation and Outer Context, include stigma and harassment. Questions exploring physician attitudes toward and experiences with abortion, including stigma and harassment, were measured using the validated instrument developed by Harris et al. [36, 37]. The clinician-researchers chose not to include questions pertaining to Greenhalgh’s domain “Implementation” at baseline and opted to explore this construct in 6-month and 12-month follow-up surveys.

**Table 1:** Example of mapping questions to domains of Greenhalgh (“Outer Context” domain shown below)

Greenhalgh’s aspects	Question	Response options	Aspect				Factor	
			Regulatory federal	Health system provincial	Health services community facility	Practice community attitudes	Facilitator	Barrier
Incentives and mandates	Have you previously ordered and stocked at your office or facility any medications for sale to your patients? (samples not applicable)	Yes No	X				National regulatory support for abortion (CMA code of ethics)	MD office to buy and stock medication
Inter-organizational norm setting and networks	Who will be responsible for ordering mifepristone at your facility?	I will be The medical director or another physician A pharmacist at our facility or in our community A non-physician, non-pharmacist staff member No one will take responsibility under current regulations Uncertain at this time [please elaborate] Other [please elaborate]		X			Provincial regulatory support for abortion (licensing)	Physician office to buy and stock medication
	Will you travel to provide medical abortion? (i.e., outside the community where you primarily practice)	Yes No			X			
	If “yes” to 19, how far is the community where you will provide medical abortion from your home?	[number] km			X			
	If “yes” to 19, how will you travel?	Road Ferry Air			X			
	Please indicate the number of women per month you currently see for abortion who travel for 3 h or more to reach your service.	[number]			X			Distance between woman and abortion service
	Are there abortion services currently available in your community?	Surgical abortion Medical abortion Both Neither			X		Established community or facility mandate	The community may have no prior abortion services

### Phase 2: Content validation

#### Participants

Physician experts in sexual and reproductive health [group II] were invited to participate in group interviews to examine face and content validity of the first version of the survey instrument developed by group I, using a modified Delphi method [38]. Group interviews took place over a 5-week period in January and February 2016. Criteria for inclusion were holding a current professional license to practice as a physician in at least one Canadian jurisdiction and willingness to participate. Emailed invitations were circulated via professional sexual and reproductive health care networks, among physicians who were current abortion providers and also among primary care and specialist physicians known to be involved in women’s reproductive health care who might consider providing abortion in their practice. A follow-up email was sent out 7 to 10 days after the first contact. Panelists in group II were encouraged to invite eligible colleagues to participate through a snowball sampling approach. We received 28 replies, and each participant received a copy of the consent form in advance and had at least 1 week to decide if they wished to participate. There were 3 panelists who were unable to attend in-person or through teleconferencing. Each of the 25 final panelists received a $200 honorarium for their 2.5 h of time.

#### Data collection

The Delphi method is a process that structures communication among large groups of individuals to collectively obtain an expert opinion for a valid and useful result [39]. The Delphi approach is used widely in research where the research problem “does not lend itself to precise analytical techniques but can benefit from subjective judgments on a collective basis” [40]. This approach enabled us to involve a representative panel of expert abortion practitioners, non-provider physicians, and physician-researchers from across the country, stratified by province, to discuss and validate the preliminary survey questions. The Mifegymiso® product label and associated regulations were publicly available, but not widely disseminated as the product was not yet on the market. Thus, prior to focus group data collection, panelists had minimal knowledge of how mifepristone was likely to be implemented in Canada.

A distinct characteristic of the Delphi method is the sequential, staged approach, whereby panelists are involved in subsequent “rounds” [41]. We modified this approach by involving group II in just one round because the goal of our group interviews was to collect important input and not to reach consensus. Furthermore, between each group interview, we revised the survey instrument to reflect the input provided by the previous group. A final version of the survey instrument was created after all five group interviews. Each group consisted of three to six expert panelists led by at least two experienced moderators who were research team members (WVN, RR, SD). Group interviews were conducted face-to-face or through video conference and began with a brief overview of the proposed regulation for mifepristone in Canada as well as an introduction to the research project. First, discussions guided by a semi-structured interview guide (see Table 2) were audio-recorded and then transcribed. Using a modified Delphi approach, drafts of the survey were distributed for expert opinion and review during each group interview. Expert panelists [group II] reviewed each question on the first version of the survey instrument for clarity and relevance. Survey questions and response options were presented individually on a PowerPoint slide. Panelists were invited to give feedback on the question wording and the rationale for their suggestions, suggest non-essential questions to be removed, provide feedback on whether or not the proposed questions would capture addressable barriers and facilitators for dissemination, and suggest appropriate sub-dimensions and items to ensure that the questions captured relevant elements of our conceptual frameworks and provided appropriate response options. We allowed time for open-ended discussion for panelists to share their perceptions, attitudes, and beliefs regarding barriers and facilitators to mifepristone implementation, including identification of additional factors that should be explored in the survey. Panelists were given the option to submit written comments to maintain anonymity. Questions that were more challenging to articulate were presented first in each group. Between group interviews, questions were refined to ensure we were adequately capturing information relevant to different practices and communities in a logical progression. The audio recording from each group interview was transcribed digitally by a transcriptionist adhering to confidentiality.

**Table 2:** Focus group instructions for question analysis

Prompts for individual items in the questionnaire	
1. How did you find the wording of the question?	
2. What are your thoughts on the purpose of the question? Elaborate if necessary: “purpose” as in “what is the question trying to ask?”	
3. What are your thoughts on the correlation between the question and the options listed for that question?	
4. Were there any options that you would like to have responded to but were not listed in the question? If so, what were these options?	
5. Were there any options that you feel were unnecessary? If so, what were these options?	
After all items from the questionnaire have been completed, the panelists will engage in a general feedback section comprised of the following questions.	
Prompts for general feedback for the questionnaire	
1. What were the strengths of the questionnaire? What were the weaknesses of the questionnaire?	
2. Was the questionnaire presented in a logical manner? If not, what would be a more logical progression for the questionnaire?	
3. Were there any missing topics of questions that you feel may be beneficial for our study? Please elaborate.	
4. If you were requested to complete this survey in the community, are there any barriers that would prevent you from completing the survey?	
5. Other comments?	

#### Analysis

Our team facilitators (WVN, RR, SD) reviewed the group interview transcripts several times independently to identify emerging themes. Together, they conducted a content analysis using the data collected from the group interviews in a recursive fashion to identify common and conflicting viewpoints in a stepwise analysis. Similarities and differences across the sub-groups (i.e., rural versus urban practice setting, specialty practice versus primary care, current abortion providers versus potential new providers) were also explored. The aim of our analysis was to ensure that questions of the survey instrument were relevant, understandable, provided clear answers, and included the range of relevant factors related to physician initiation and ongoing provision of mifepristone medical abortion practice.

##### Step 1: Usability

Step 1 of the analysis involved an iterative process of identifying and itemizing potential changes to the format of the survey questions. This included a thorough review of comments from the group interviews about which survey questions were clear, concise, and comprehensive, and which questions required additional editing. As Fowler [38] suggests, “Poor question design is pervasive, and improving question design is one of the easiest, most cost-effective steps that can be taken to improve the quality of survey data” (p. vii). For example, each focus group provided feedback on the importance of consistency between Likert scale rating response directions throughout the survey, i.e., from strongly disagree to strongly agree, from less to more, from harmful to beneficial, or from extremely difficult to extremely easy. Other discussions involved clarifying ambiguous terms or concepts and the potential consequences of response alternatives for participant answers. For example, to understand the degree of burden a physician will experience if they are required to sell mifepristone directly to patients at their facility, we initially asked about the cost of stocking the medication. However, two key concepts in this question were identified: “burden” and “cost.” We therefore went on to distinguish both “cost” burden and the “administrative” burden of ordering and maintaining stock. Panelists employed at private clinics suggested they would be considering the high purchase cost as part of the overhead of keeping stock (estimated at $300 per dose). Conversely, panelists employed in settings that already sell contraceptives reported that they would experience no burden themselves, but that it was likely their patients would, due to the high cost.

##### Step 2: Content and context

The second step of the analysis involved a general inductive approach to understand the necessary content modifications to the questions and response options. For example, each focus group engaged in the discussion about the definition of the term “community.” A participant employed in a rural setting explained that “community” does not accurately represent the vast geographic population catchments that rural and remote physicians typically serve: “I’m looking at women that are a two-to-three-and-a-half-hour flight away from me, that are in my ‘service region’” (focus group 2, family doctor, rural). Conversely, a participant employed in an urban setting stated, “If I have to drive for more than 10 minutes, I’m outside of my community” (focus group 4, OB-GYN, urban). As poorly defined terms can negatively impact the reliability of participants’ responses, we edited and replaced ambiguous terms or jargon based on panelist feedback. This included defining terms in the wording of the question or asking supplementary questions (e.g., *How far from your home is the community where you will provide medical abortion?*).

Several additional questions were suggested by the panelists that did not contribute to the goals for the study or fit within the survey’s conceptual framework. For example, panelists suggested to include questions related to patient satisfaction or to conduct an economic analysis comparing surgical abortion to mifepristone medical abortion. These questions were not included to ensure the final survey met the aims of the research question [26]. Following all group II input and the associated analysis, a second version of the survey instrument was iteratively reviewed by group I until consensus was reached and the indicated domains in the theoretical frameworks were represented by appropriate questions.

##### Step 3: Thematic analysis of facilitators and barriers

In order to ensure that our survey instrument captured relevant perceived barriers and facilitators to implementation of mifepristone, we also conducted a thematic analysis of group interview transcripts, informed by critical realist principles—that is, we explored how panelists made meaning of their abortion provision intentions and experiences and the ways that social context informed them, while considering the material reality in which mifepristone would be implemented in Canada [42]. The purpose of conducting a thematic analysis of the group interview data was to enhance the development of the survey instrument. One qualitatively trained researcher (CD) read the transcripts to become immersed in the data and identify patterns across panelists and groups. She independently developed a codebook of initial categories related to panelists’ perceptions of barriers and facilitators to implementation of mifepristone, following these phases: (1) initial coding for candidate categories and patterns in the transcripts, (2) focused coding for main categories and patterns, and (3) reviewing and refining categories. This nurse-researcher was not involved in the data collection phases, which helped to facilitate a data-driven inductive approach as she did not share the same assumptions and analytic preconceptions as to the other members of the research team, who were experts in family planning research. She coded the transcripts independently and discussed her analysis at weekly intervals with a postdoctoral fellow (SM) with expertise in qualitative methods who had been involved in designing the protocol and data collection. Together, they engaged in a reflexive dialog about the emerging categories, the significance of the categories, the convergence and inconsistencies between the groups [43], and the consequent implications for facilitating or impeding access to mifepristone medical abortion. Together, they wrote the results of the analysis into a rich, descriptive narrative. All analysis was conducted in Microsoft Word.

#### Finalizing the survey procedure

The second version of the survey instrument was directly translated in French using a translation service. The Francophone investigators on our team (EG, MSW) reviewed the translated survey for content and face validity to ensure the correct translation of medical terminology and relevance for Francophone physicians.

Following the translation, the survey instrument was entered, in both English and French, into a Research Electronic Data Capture (REDCap) platform, a secure web-based meta-data-driven application, housed at our research institution [44]. Branching logic was used to determine the respondents’ pathway in the survey. This mitigated the potential for survey fatigue by ensuring questions were relevant based on variables in responses.

### Phase 3: Pilot testing

To assess usability and reliability of the online survey, we invited the 9 physician co-investigators on the wider study, who were not involved in phase 2 content validation, to pilot test the second version of the survey instrument [group III]. To support rigor, conducting a pilot test with participants who are representative of the study sample interests is recommended [38, 45]. Each physician co-investigator was emailed a link to the REDCap survey in December 2016 to January 2017 and was given instructions to complete the survey and respond by email with recommendations. Our team revised the online survey in real time as recommendations from group III were received. This iterative cycle of testing, revising, and retesting the survey identified fewer issues in each subsequent co-investigator response until no further issues remained. The English survey link was tested 139 times and the French survey link was tested 43 times among group III, and finally by all members of group I, during this phase.

## Results

### Development of preliminary survey items (phase 1)

The first version of the survey instrument consisted of 68 questions involving demographic and practice characteristics (22 questions), previous experience providing abortion services (8 questions), theoretical domains from Greenhalgh’s framework (24 questions), and the 12-item questionnaire adapted from Légaré’s validated instrument [27]. The Légaré instrument questions applied to a range of content areas; therefore, very few wording changes were required (see Table 3). Appropriate questions stemmed from 3 previously developed and fielded instruments developed by CART for the diffusion of contraception practice innovations [34, 35, 46] with minor modifications (e.g., “mifepristone” replacing “contraceptive” where appropriate).

**Table 3:** Continuing professional development questionnaire (adapted from Légaré et al. [33])

1	I intend to provide medical abortion.	Strongly disagree			Strongly agree	
		1	2	3	4	5
2	To the best of my knowledge, the percentage of my colleagues who provide medical abortion is:	0–20%, 21–40%, 41–60%, 61–80%, 81–100%				
3	I am confident that I could provide medical abortion if I wanted to.	Strongly disagree			Strongly agree	
		1	2	3	4	5
4	Providing medical abortion is the ethical thing to do.	Strongly disagree			Strongly agree	
		1	2	3	4	5
5	For me, providing medical abortion would be:	Extremely difficult			Extremely easy	
		1	2	3	4	5
6	Now think about a co-worker whom you respect as a professional. In your opinion, does he/she provide medical abortion?	Never			Always	
		1	2	3	4	5
7	I plan to provide medical abortion.	Strongly disagree			Strongly agree	
		1	2	3	4	5
8	Overall, I think that for me providing medical abortion would be:	Useless			Useful	
		1	2	3	4	5
9	Most people who are important to me in my profession provide medical abortion.	Strongly disagree			Strongly agree	
		1	2	3	4	5
10	It is acceptable to provide medical abortion.	Strongly disagree			Strongly agree	
		1	2	3	4	5
11	I have the ability to provide medical abortion.	Strongly disagree			Strongly agree	
		1	2	3	4	5
12	Overall, I think that for me providing medical abortion would be:	Harmful			Beneficial	
		1	2	3	4	5

#### Results of expert content validation (phases 2 and 3)

Expert panelists [group II] in phase 2 (*n* = 25) who participated in a total of 5 group interviews represented physicians who provided abortion and those who had not, from primary care and specialty practice settings, rural and urban communities, in 3 provinces and 2 territories (British Columbia, Ontario, Nova Scotia, North West Territories, and Yukon) including those trained as family physicians and obstetrician gynecologists (see Table 4). After phase 2, 5 clinical content experts (which included representation from the fourth province, Quebec) finalized the Mifepristone Implementation Survey to distribute for pilot testing. Group III participants involved in the final phase of development provided representation from additional 3 provinces (Alberta, Manitoba, and Quebec). By phase 3, the survey length increased from 47 baseline questions to 65 baseline questions which addressed Greenhalgh’s domains: “System Readiness for Change” (*n* = 19), “Outer Context” (*n* = 21), and “System Antecedents for Innovation” (*n* = 25). This increase was as a result of feedback received during phase 2, in which expert panelists [group II] identified several questions where division into 2 individual questions improved clarity. For example, a participant suggested, “I guess maybe the question is, you might have one [an ultrasound] in your facility but if you’re not trained to use it, do you have somebody who can do it?” (focus group 4, OB-GYN, urban). This is an example of how questions related to practical implications and to constructs from Greenhalgh’s framework [24]. For this participant, it was more meaningful to ask about *access* to a skilled ultrasonographer as a potential barrier rather than if they had an ultrasound machine in their facility.

**Table 4:** Characteristics of focus group panelists (group II)

	Focus group 1 (*n* = 5)	Focus group 2 (*n* = 6)	Focus group 3 (*n* = 5)	Focus group 4 (*n* = 6)	Focus group 5 (*n* = 3)	Total, *n* (%)
Discipline						
Family practice	3	4	3	2	2	14 (56%)
Obstetrics-gynecology (OB-GYN)	2	2	2	4	1	11 (44%)
Experience* (years)						
No abortion experience	1		1		1	3 (8%)
Less than 5		1	1	1	1	4 (15%)
5–9	1		1			2 (13%)
10–19		2	2		1	4 (15%)
20+	3	2		1		6 (24%)
Primary practice type						
Hospital	2	2	1	3	2	10 (40%)
Clinic	2	1	1	3	1	8 (32%)
Primary care		2	3			5 (20%)
Gender						
Male		2	2	6		10 (40%)
Female	5	4	3		3	15 (60%)
Province						
British Columbia	5	4	5			14 (56%)
Ontario				6	2	8 (32%)
Nova Scotia					1	1 (4%)
Territories		2				2 (8%)
Setting						
Urban	5		3	6	2	16 (64%)
Rural		6	2		1	9 (36%)
Total						25 (100%)

*Experience refers to the number of years the participant has been providing abortion services (medical, surgical, or both) after post-graduate training; numbers may not add up to 100% due to incomplete data

Consideration of rural and urban differences was key in validating response options. For example, open-ended responses suggested that practitioners in rural settings defined community very broadly in comparison with practitioners in urban centers: “I think 15 kilometers is a pretty urban number” (focus group 1, OB-GYN, rural). Similarly, practitioners employed in hospital-affiliated abortion facilities were less concerned about ordering, stocking, or selling medications than their colleagues in private offices or clinics: “Our hope is that it will be on our hospital formulary and that it be at no cost to the patient” (focus group 5, family physician, urban).

#### Perceptions of potential barriers and facilitators to mifepristone implementation

Analysis of group interviews led to our identification of categories that represent physicians’ and abortion experts’ perceptions of potential barriers and facilitators to the implementation of mifepristone in Canada. None of the categories discussed was characterized as facilitators; the key categories that emerged from the analysis of the group interview transcripts included the following barriers: *physicians dispensing the medication*, *mandatory training to become a prescriber*, *burdens for patients*, *lack of remuneration for mifepristone provision*, and *services available in my community*. This qualitative data further supported and enriched the expert content validation by clarifying and validating the questions and response options.

##### Burdens for patients

Group panelists (group II) emphasized that having medical abortion services in primary care in remote communities would be important to ensure that patients can remain close to home. Rural physicians from one focus group identified potential revisions to the survey to reflect the reality of providing family planning services in remote communities that serve vast catchment areas and have limited resources for surgical backup in the event of a failed first-trimester medical termination. As one physician from the far north clarified, a question could explore, “If I’m in a bigger [rural] community like I am, I am the abortion provider, ‘What communities do [you] service and how far [away] are the women that are potentially seeking that help?’” She went on to explain that her large community serves “women that are a 2 to 3 ½ hour flight away” (focus group 2, family doctor, rural). Panelists also perceived that the federal requirement for patients to sign mandatory consent forms and be observed while ingesting the medication was “so insulting to women” (focus group 1) and excessive in comparison with other treatments: “I suspect you sign fewer forms if you’re going for cardiovascular surgery” (focus group 1).

##### Physicians dispensing the medication

Panelists observed that the federal restriction for “physician-only (not pharmacist) dispensing” of mifepristone would be a key barrier in *primary care* settings, compared to purpose-built abortion facilities or hospitals. The requirement that a physician dispenses medication in place of the medication being available to purchase at a pharmacy is highly unusual in a Canadian context. This would require physicians interested in prescribing mifepristone, to set up the infrastructure to stock and sell the medication: “So it’s sort of a two-pronged thing. One is additional cost associated with the actual selling of the prescription to the patient like a dispensing fee, whatever. The other is capital cost to be able to sell it. So the cabinet maker, and the fees for the credit card machine” (focus group 4, OB-GYN, urban). Most physicians’ offices do not have such infrastructure, which led panelists to engage in discussion about what, hypothetically, this new practice change might look like: “And this is a lot of money. Like what would people do? Take VISA? I mean it does raise logistical questions, right?” (focus group 4, family physician, urban). Physician-only dispensing also may prevent patients from filling their prescriptions at a convenient time and location: “That in itself is a burden, if you have to wait for the pharmacy to courier it back [to the physician]. You’ve only got a certain time. Are you going to put her out in the waiting room and see someone else or?” (focus group 3, OB-GYN, rural, experienced provider).

##### Mandatory training to become a prescriber

In Canada, physicians are expected to maintain skills and competency through self-study and continuing professional development. Panelists’ comments indicated that the unique requirement of completing a mandatory training program, only for this medication, and to register with the manufacturer may discourage physicians and pharmacists to adopt medical abortion practice and patients would have limited access to qualified providers. As one participant described: “But if you go in there and the [trained] person isn’t working that day, or has to be sick, or on holidays, or anything else and nobody else can fill that stupid prescription?” (focus group 3, family physician, urban). It also may mean that trained physicians would face the burden of being a solo abortion provider: “I can’t see a bunch of people doing all the training. Like, I anticipate it’s going to put more of a burden on smaller numbers of people.” (focus group 2, family physician, rural).

##### Lack of remuneration for providing medical abortion

The lack of existing remuneration structures (e.g., billing codes) for medical abortion was identified as another critical barrier. As one experienced abortion provider clarified, providers would have to cobble together different fees to adequately cover the costs of providing this service: “As you say, if somebody does not, they will bill whatever they can do in the way of a counselling fee, a physical examination, et cetera, and so the question is, ‘Is that going to be adequate?’ So, if we’re going to do it, you might want to just say straightforwardly, ‘In your jurisdiction, are you paid well enough to cover your costs?’” (focus group 1, family physician, urban). She and other experienced providers noted that low fees for medical abortion could be a deterrent for new providers and their practices: “But my point is, automatically there’s a cost. It’s whether it’s enough of a barrier for you to provide it or not.” (focus group 3, family physician, urban).

##### Services available in my community

Finally, panelists’ feedback illuminated the barriers associated with having limited resources to support the provision of medical abortion. Timely access to ultrasound was a particular challenge for small rural communities, as one participant described: “Yeah, so I mean in smaller towns ultrasound is not – it’s really – we have a bedside ultrasound in Emergency, but there’s no other ultrasound in town available outside of the hospital. Then in [larger town] there’s community ultrasound place, but they’re not generally run – You know, they’re like business hours right” (focus group 5, family physician, rural). Further, in both urban and rural sites, some panelists described having limited support from surgeons in the rare event of a failed termination or complication that requires surgery. While colleagues may provide support if “the person is unstable or it’s an emergency, or they have to, ‘those’ people who don’t want to be involved in terminations will choose not to be involved” (focus group 3, OB-GYN, rural). Limited collegial support can be a deterrent for new abortion providers, who may not wish to strain their interprofessional relationships by introducing abortion to their scope of practice.

#### Results of pilot testing (phase 3)

In phase 3, 1 round of surveys was administered via REDCap and distributed for readability and usability testing through an online link that included modifications based on the feedback from the larger CART-GRAC network (group III). The second version of our survey instrument consisted of 9 demographic questions, 52 questions mapped to Greenhalgh’s conceptual model (see Table 1 for an example), the 12-item questionnaire adapted from Légaré’s validated instrument, and 17 open-ended questions that provided respondents with the option to elaborate their responses. During phase 3, minor technical changes took place including spelling errors, changing the order of demographic questions, improving the clarity of partner logo images, and moving hyperlinks to resources to the end of the survey. Once each of the technical changes was addressed, we established our final survey instrument.

Between phases 2 and 3 of the Mifepristone Implementation Survey Development Study, Health Canada removed the requirement for physician observation of the patient swallowing mifepristone (October 2016, prior to market availability in January 2017). This change was due in part to the early dissemination of our focus group finding that observation would create an unnecessary burden for patients [47–49]. However, package labeling in the marketed product did not reflect this change until September 2017. Our research team made the decision to include the questions we had developed and tested in focus groups around physician observed dosing to capture the impact of a regulatory change not necessarily reflected physically on the packaging materials and on physician knowledge and practice.

## Discussion

We undertook a rigorous process to develop and pilot test a survey instrument to investigate the health policy, health system, and health care delivery factors impacting first-trimester mifepristone abortion practice in Canada. In phase 1, family planning experts grounded the first version of the survey instrument in established theoretical frameworks. In phase 2, 5 group interviews leveraged the expertise of reproductive health providers from across the country and determined that Health Canada’s proposed regulations would act as barriers to implementation, particularly in primary care. We collected initial impressions on potential barriers from a sample population of physicians and developed a 65-item baseline survey using 3 of Greenhalgh’s domains: “System Readiness for Change” (*n* = 19), “Outer Context” (*n* = 21), and “System Antecedents for Innovation” (*n* = 25) to pilot test. In phase 3, a larger network provided feedback to refine the survey. The Mifepristone Implementation Survey is the result of a 3-phase process to develop a comprehensive, nationally relevant instrument. We intend to administer our survey with a national sample of mifepristone providers and to use our findings to inform future medical abortion policy, health system, and service decisions in Canada. Our larger program of research will go on to collect 6-, 12- and 24-month follow-up data from this prescriber survey, and from a planned pharmacist survey, to measure the change in practice experiences and behavior. We will tailor follow-up survey content to focus on the constructs of Diffusion of Innovations that relate to the barriers and facilitators we identify from the baseline survey and to determine the uptake of mifepristone abortion practice by respondents.

Our study process resulted in a more comprehensive survey than any we were able to identify in the international literature to understand the barriers to contraceptive and abortion service provision in high-income countries [5, 50–52]. For example, to assess similar factors in a related field in 2011, Hulme et al. conducted 72 interviews with healthcare providers and health system stakeholders to understand the barriers to contraceptive use in Canada [53]. Interviews were informed by 2 validated frameworks for access and quality in family planning; however, the sample primarily included participants from Canada’s 3 largest provinces, Quebec, Ontario, and British Columbia, and may not have reflected policy issues in other provinces, territories, and between rural and urban [54]. Furthermore, in 2013, Guilbert et al. surveyed 78 abortion facilities and their abortion providers across Canada to understand the national availability and practice of first-trimester medical abortion [5]. Their survey instrument, which was adapted from USA research for the Canadian context, investigated the facility and staff demographics as well as clinical practice such as pre-procedure evaluation, medication regimens, and follow-up for abortion service patients [51, 52]. The survey was suitable to describe the usual practice, but facilitators and barriers to first-trimester medical abortion practice were not investigated. This limitation was accounted for in our survey instrument by specifically developing questions to target barriers to medical abortion practice identified by physicians from diverse practice settings.

A significant strength in the development of this survey was the use of established conceptual frameworks that assess multiple dimensions of implementation and that explore physician and organizational behavior in implementing an innovation. While Greenhalgh’s [28] comprehensive model of diffusion, dissemination, and implementation is based on a systematic review of empirical and theoretical models, it has been criticized for its lack of “operational definitions or measurement” for many of the constructs (Cook et al. 2012, p. 12). Our survey development process is an “operationalization” of the Greenhalgh framework and applies the framework to a health system implementation scenario.

Our study was also strengthened by group interviews with a national expert panel representing both family medicine and obstetrics-gynecology training, urban and rural communities, and specialty and primary care practices. This allowed us to capitalize on the strengths of group decision-making despite geographic separation. The panelists’ diverse contributions enhanced the clarity and relevance of the questions thereby ensuring high face validity of our survey content. This exploratory approach surfaced issues that could not have been identified by our research team alone. Further, this approach allowed us to explore physicians’ perceptions of potential barriers and facilitators due to Health Canada’s restrictive regulations for prescribing and dispensing mifepristone. Physician dispensing and mandatory training were regulatory barriers that were perceived to have the potential to increase the burden on patients seeking medical abortion by inhibiting physicians from implementing mifepristone abortion care in their practices. Lack of billing codes to support remuneration and limited availability of ultrasound and surgical backup were practical barriers that were related to regional health system decision-making, rather than federal medication policy. Furthermore, Dressler et al. [54] identified barriers that practitioners experience based on their geographic location across Canada. They found that logistical barriers to provision, professional isolation, and lack of replacement providers including barriers to receive training are uniquely identified by providers in rural and remote communities. These concepts were validated by the expert panel as important areas to explore in the survey. The barriers identified in this current study are investigated in the survey instrument so that we may further understand what factors influence the implementation of mifepristone across the country.

Limitations to the Delphi approach include the potential for panelists to be influenced by the questions formulated by the research team and panelists’ failure to understand the primary purpose of the study. For example, we did not aim for our survey to capture all potential questions related to barriers and facilitators of abortion provision in Canada, but only those most relevant to mifepristone. It was challenging to balance the length of the survey with our desire to collect adequate, relevant information. This meant that we had to prioritize several of the dimensions of the framework over others, based on the relevance to our research objectives. For example, we prioritized collecting data on barriers perceived to be addressable through health policy, system, or service delivery changes not individual attitudes or knowledge change.

Phase 3 pilot testing only focused on the readability and technical aspects of the online survey instrument. We did not include specific measurements such as Cronbach alpha coefficients to assess internal consistency, test-retest reliability testing, or factor analysis to describe variability among items. However, some of the basic instruments we used to build our survey instrument did report strong psychometric testing [33].

The introduction of mifepristone and the development of new clinical practice guidelines [24] present an exceptional opportunity to improve women’s access to abortion in Canada, particularly in rural and remote areas [1, 55–57] and to improve women’s safety and privacy by reducing the stigma and harassment that can occur for women seeking a surgical abortion [58]. To advance mifepristone practice in Canada, health policy, system, and health care delivery implementation factors will need to be identified to facilitate the promotion of equitable access to abortion care. This survey will be used to assess those barriers and facilitators of mifepristone implementation in Canada. Given that the rates of mifepristone implementation have differed greatly internationally, it will be crucial that barriers to practice are adequately captured and presented to decision-makers across Canada in a timely way.

## Conclusion

The Mifepristone Implementation Survey Development Study employed a rigorous process to develop a comprehensive, nationally representative survey instrument. The specific questions were developed using frameworks that comprehensively covered barriers and facilitators to provider implementation of medical abortion practice. As such, they constitute a good basis for surveys in other jurisdictions that want to study barriers and facilitators to medical abortion. Data from the fielding of this survey will be used to inform decision-makers across Canada and to facilitate more equitable access to abortion services.

## Data Availability

The data generated and analyzed during the current study are available from the corresponding author upon reasonable request. The final version of the Mifepristone Implementation Survey is available at http://med-fom-cart-grac.sites.olt.ubc.ca/files/2019/03/Mife-MD_Survey.pdf
